# In Vitro Characterization of Indigenous Probiotic Strains Isolated from Colombian Creole Pigs

**DOI:** 10.3390/ani10071204

**Published:** 2020-07-15

**Authors:** César Betancur, Yordan Martínez, Guillermo Tellez-Isaias, Mavir Carolina Avellaneda, Borja Velázquez-Martí

**Affiliations:** 1 Departamento de Ciencias Pecuarias, Facultad de Medicina Veterinaria y Zootecnia, Universidad de Córdoba, Montería 230002, Colombia; betanci@yahoo.com; 2 Science and Agricultural Production Department, Zamorano University, Francisco Morazán P.O. Box 93, Honduras; ymartinez@zamorano.edu (Y.M.); cavellaneda@zamorano.edu (M.C.A.); 3 Department of Poultry Science, University of Arkansas, Fayetteville, AR 72701, USA; gtellez@uark.edu; 4 Departamento de Ingeniería Rural y Agroalimentaria, Universitat Politècnica de València, Camino de Vera s/n, 46022 Valencia, Spain

**Keywords:** lactic acid bacteria, native pig, probiotic, Zungo Pelado

## Abstract

**Simple Summary:**

In the pig industry (mainly after weaning), enteric diseases are frequent associated with pathogenic bacteria such as *Salmonella* spp. and *E. coli*. Although the European Union has banned the use of growth-promoting antibiotics, many countries use these synthetic medications with widespread, which has led to an increase in the number of antibiotic-resistant pathogens, cross-resistance, and bioaccumulation in tissues. Probiotics are beneficial bacteria that live in the intestine and improve the host health; they are also one of the main alternatives to subtherapeutic antibiotics. Therefore, our goal was to obtain lactic acid bacteria from Colombian creole pigs that had never consumed a medicated diet and to carry out in vitro probiotic tests. Three strains lived before were identified: *L. plantarum* CAM6, *L. plantarum* CAM7 and *L. plantarum* CL4. The obtained strains have good activity in the physiological, antibacterial and antibiogram tests. Further studies to evaluate the efficacy of this strain in commercial pigs are currently being evaluated.

**Abstract:**

Three lactic acid strains were isolated from feces of the native Zungo Pelado breed of pigs (*n* = 5) and presumably identified as belonging to the *Lactobacillaceae* family by morphological techniques showing that they were Gram-positive/rod-shaped and catalase- and oxidase-negative. They were then identified by biochemical tests using API 50CHL as *Lactobacillus plantarum* (CAM6), *Lactobacillus brevis* (CAM7), and *Lactobacillus acidophilus* (CL4). However, 16S rRNA identification showed that all three strains were *Lactobacillus plantarum*. Additionally, all three isolates were able to grow in pH 3 and 4. Interestingly, the growth of the CAM7 strain decreased at pH 5.6 compared to that of the CAM6 strain (*p* < 0.05), and the growth of the CL4 strain was reduced at pH 7(*p* < 0.05). All three candidates showed good growth on bile salts (≥0.15%), and CAM6 and CAM7 showed better tolerance at higher concentrations (0.30%). Similarly, all strains tolerated sodium chloride (NaCl) concentrations from 2 to 10%. These strains also grew well at all temperatures tested (30, 37, and 42 °C). The CAM6 strain showed in vitro antibacterial activity against selected enteropathogenic bacteria (*Escherichia coli* strain NBRC 102203 and *Salmonella enterica* serovar Typhimurium 4.5.12) and commensal bacteria (*Klebsiella pneumoniae* ATCC BAA-1705D-5 and *Pseudomonas aeruginosa* ATCC 15442) and resistance to all antibiotics except amoxicillin. Further studies to evaluate the effects of these probiotic candidate strains in commercial pigs are currently underway.

## 1. Introduction

The indiscriminate use of antibiotics for disease prevention and as growth promoters in animal husbandry increases the number of antibiotic-resistant pathogens [1] and the risk that these resistant pathogens can be transmitted to humans [2]. Moreover, the presence of antibiotic residuals in meat products might pose a significant public health risk as, once consumed, antibiotic residuals might also increase bacterial resistance in humans [3]. Some of these pathogens have proven to be extremely resistant to all commonly used antibiotics and/or capable of rapidly developing resistance when exposed to antibiotic prophylaxis or treatment [4].

Antibiotics are generally ineffective in the treatment of multidrug-resistant bacteria. The incidence of foodborne pathogens, such as *Salmonella* spp., *Escherichia coli,* and *Campylobacter* spp., is increasing worldwide, with reports of antibiotic resistance in clinical isolates of these and other enteric pathogens [1]. The diminishing effectiveness of antibiotics—wonder drugs of the 20th century—has become a looming threat to public health. In response, on 1 January 2017, the United States Food and Drug Administration, working to ensure the judicious use of antibiotics in human medicine, restricted the employment of such antibiotics in growth promotion and expanded the list of feed-grade antibiotics classified as Veterinary Feed Directive drugs [5]. Equally concerning is the fact that indiscriminate use of antibiotics can induce disruption of the intestinal microbiome, reducing the production of short-chain fatty acids and increasing luminal pH in the distal gastrointestinal tract, which results in dysbacteriosis [6]. 

In recent years, substantial scientific evidence has shown that the use of certain antibiotics increases enteric colonization of antibiotic-resistant strains of enteric pathogens in domestic animals [7]. In the swine industry, enteric infections are frequent causes of diarrheal diseases and represent a serious health threat to the animals. Infections can occur at any point in their lifetime, but the highest incidence of disease occurs during certain critical times, which can be divided according to the production system into the suckling, weaning, and growing periods. 

During the first week of life, piglets commonly encounter infections from *Clostridium perfringens* type A or enterotoxigenic *E. coli* [8,9]. To date, several compounds have been used as alternatives to antibiotics, such as organic acids, probiotics, prebiotics, and plant extracts. De Mille et al. [10], Chen et al. [11], and Julio-Gonzalez et al. [12] have demonstrated favorable results of the use of such alternatives in terms of both health and productivity [13]. However, a major strategic concern is the lack of knowledge of the mechanisms through which the alternative substances can protect intestinal cells from the damage induced by pathogens [14].

Still, recent global research has indicated the use of probiotics to be a promising alternative to antibiotic growth promoters [15,16]. The high acidity in the stomach and great concentration of bile components in the proximal intestine are the first host factors that may affect commercial probiotics [6,17,18]. 

Although probiotics are commonly used, only in the last two decades have scientists discovered the physiological mechanisms behind the improvement in health and performance of animals in the meat production industry [19,20]. Several investigators have demonstrated the beneficial effects of probiotics, such as modulating the host’s immune response (both the innate and adaptive response) [21], stimulating immunoglobulin production (Ig) (mainly IgA by B lymphocytes [22]), promoting the animal’s response to external aggressors [23], exerting antioxidant properties [24], producing enzymes, and reinforcing the integrity of the intestinal barrier through the phosphorylation of proteins from the narrow unions of intestinal epithelium cells [25].

Many microorganisms have been used as probiotics, but lactic acid bacteria (LAB) have been shown to be the most effective probiotic agents in swine production [1,6,26]. Several studies confirm the beneficial effect of these bacteria on health and productivity in swine production [27,28]. In this sense, Wang et al. [29] showed that strains of *Lactobacillus plantarum* ZLP001 isolated from the gastrointestinal tracts of healthy pigs modulate their gut microbiota. As the natural microflora stabilizes in the gut easily, and quickly propagates to one specific species [27], an isolate from the host itself will be a more effective probiotic than isolates from other sources.

The present study was conducted with the objective of phenotypically and phylogenetically characterizing the lactic acid bacteria isolated from the gastrointestinal tracts of the native Zungo Pelado breed of pigs in the northern coastal region of Colombia and for the in vitro evaluation of these strains as possible probiotic candidates that could be used as natural growth promoters to replace subtherapeutic antibiotics in swine production.

## 2. Materials and Methods

The present experiment was conducted in the Biotechnology Laboratory, Faculty of Sciences, GRUBIODEQ group of the University of Córdoba, Montería, Córdoba, Colombia.

### 2.1. Bacterial Isolation

Five pigs (Zungo Pelado) aged 6 months and weighing 55 ± 5 kg were randomly selected from the AGROSAVIA Conservation Center, Cereté, Córdoba, Colombia. The pigs were fed commercial feed (without antibiotic growth promoters) and local agricultural byproducts, such as liquid processed feeds and feeds derived from the citrus and sugarcane industries. It is important to note that the veterinarian of the AGROSAVIA Conservation Center, Cereté, Córdoba, Colombia, certified that these pigs were apparently healthy since no symptoms, signs, or pathologies associated with any diseases were found and the blood and feces analyses were normal.

Stool samples were taken directly from the rectum with disposable gloves. Samples, in groups of three, were placed with ice packs in hermetic containers and transported to the laboratory. One gram of a sample from each pig was mixed with 9 mL of peptone water and shaken in a vortex mixer at 150 rpm for 15 min, and serial dilutions in sterile saline solution 0.9% were plated into de Man, Rogosa and Sharpe (MRS) agar plates (Catalog No. 288110, Becton Dickinson and Co., Sparks, MD, USA). Plates were incubated anaerobically (carbon dioxide atmosphere) for 48 h at 37 °C.

### 2.2. Morphological and Biochemical Tests

Individual isolates were tested for oxidase production, catalase, and Gram stain affinity. Colonial characteristics were observed on MRS agar, and cell morphology was observed with a microscope.

### 2.3. Bacterial Identification

The selected strains were initially identified using the API 50CHL medium and API 50CH, according to the manufacturer’s instructions (BioMérieux, Marcy l’ Étoile, France), and the results were interpreted by the software APIWEB (BioMérieux). The biochemical identification of each strain was confirmed by the sequencing of the 16S rRNA gene. Genomic DNA was extracted from isolated colonies in MRS using a commercial Power Soil DNA Isolation Kit (QIAGEN, US), according to the manufacturer’s instructions. The 1465 bp region of the ribosomal gene 16S was amplified by polymerase chain reaction (PCR) using universal primers F27 (5′-AGAGTTTGAT CMTGGCTCAG-3′) and R1492 (5′-TACGGYTACCTTGTTACGACTT-3′), and its fragments were purified. Once purified, the products were sent to a reference laboratory for sequencing (Macrogen Inc., 2017, Seoul, Korea). The sequenced products were evaluated using the Basic Local Alignment Search Tool [30].

### 2.4. Resistance under Gastrointestinal Conditions: Temperature, pH, and Sodium Chloride

In this series of in vitro studies, a basal MRS medium was used. An overnight culture of each isolate was used as the inoculum. The cells were centrifuged and resuspended in 0.9% sterile saline. The three inoculums (100 μL each) were suspended in 10 mL of MRS agar in test tubes. The incubation timepoint was recorded at 24 h for evaluation of the variables (pH, sodium chloride (NaCl), and temperature). The temperatures tested were 30, 37, and 42 °C. The sodium chloride (NaCl) concentrations were 2, 4, 7, and 10% (*w*/*v*). The isolates were tested for survival at four pH levels: 3, 4, 5.6, and 7. The tubes were incubated with reciprocal shaking at 37 °C for the pH and NaCl tests. The turbidity of each tube was determined by measuring optical density (OD) at 600 nm with a spectrophotometer (Spectrophotometer UV-1800; Shimadzu, Japan). Then, each isolate was streaked onto MRS agar to check for the presence or absence of growth in order to confirm the survivability of each strain. Each treatment was tested with triplicate tubes.

### 2.5. Bile Salt Tolerance

The method of Latorre et al. [31], with some modifications, was used to determine bile salt tolerance. The MRS agar tubes containing 0.05, 0.10, 0.15, or 0.30% bile salts (Catalog No. 213010, Becton Dickinson and Co., Sparks, Maryland, USA) were inoculated with 10^6^ CFU/mL of each probiotic strain, after being centrifuged at 3000× *g* for 15 min and washed three times following overnight growth. Samples were incubated for 24 h at 37 °C with shaking at 100 rev./min. Growth in control (no bile salts) and test cultures was evaluated at 24 h by streaking samples onto MRS agar for the presence or absence of growth.

### 2.6. Assessment of Antimicrobial Activity against Enteropathogenic Bacteria

The lactic acid isolates were screened for in vitro antimicrobial activity against *Escherichia coli* strain NBRC 102203, *Salmonella enterica* serovar Typhimurium 4.5.12, *Klebsiella pneumoniae* ATCC BAA-1705D-5, and *Pseudomonas aeruginosa* ATCC 15442. Ten-microliter aliquots of 10^−6^ to 10^−7^ dilutions of each tested isolate were placed in the center of MRS plates. After 24 h of incubation at 37 °C, the plated samples were coated with Tryptic Soy Agar (TSA) (Catalog No. 211822, Becton Dickinson, Sparks, MD, USA) containing 10^6^ CFU/mL. After 24 h of incubation at 37 °C, the plates were evaluated and the colonies that produced zones of inhibition were selected.

### 2.7. Antibiotic Susceptibility Testing

Antibiotic susceptibility was determined using the disk diffusion method [32]. The probiotic candidate and pathogenic strains were subjected to different antibiotics commonly used to treat enteric and respiratory infections in swine production: ciprofloxacin 5 µg, dicloxacillin 1 µg, trimethoprim sulfamethoxazole 25 µg, amoxicillin 10 µg, tetracycline 30 µg, and doxycycline 5 µg. The individual strains were inoculated into MRS agar plates, and the pathogenic strains were inoculated into Mueller–Hinton agar plates for the antibiotic susceptibility test. After inoculation, the antibiotic disks were placed onto the surface of the cultures. All plates were incubated at 37 °C for 24 h under microaerophilic conditions. After incubation, the diameter of the zone of inhibition was measured. A diameter of ≥21 mm indicated a susceptible (S) strain, a diameter of 16–20 mm indicated an intermediate (I) strain, and a diameter ≤15 mm indicated a resistant (R) strain [33].

### 2.8. Data and Statistical Analysis

Variance analyses were performed to verify significant differences between the means with a significance level of *p* < 0.05. The Duncan test was used to describe the differences between strains (*p* < 0.05). All statistical analyses were completed using SPSS software (SPSS Inc., IBM Corporation, Version 22, Chicago, IL, USA). The counts of viable microorganisms were transformed to Log N to ensure normal conditions in the growth curve.

## 3. Results

### 3.1. Morphological and Biochemical Tests of the Lactic Acid Bacteria Isolates

**Figure 1:**
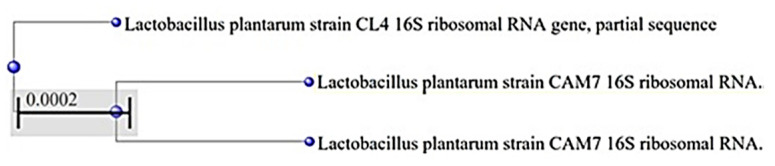
Phylogenetic tree of CAM6, CAM7, and CL4. This distance gives us a 0.0002% dissimilarity between the CL4 strain and the CAMs.

All three isolated strains were Gram-positive microorganisms with rod morphology, occurring singly or grouped in short chains, with catalase- and oxidase-negative characteristics of lactic acid bacteria. API 50CHL identification provided a different name for each of the three selected strains: *Lactobacillus plantarum* for CAM6, *L. brevis* for CAM7, and *L. acidophilus* for CL4 (Table 1). However, 16S rRNA identification showed that all three strains were *Lactobacillus plantarum* (Figure 1).

### 3.2. Physiological Characteristics of the Lactic Acid Bacteria Isolates

All three isolates tolerated pH 3 and 4, and the CAM7 strain showed a significant reduction (*p* < 0.05) in growth at pH 5.6 when compared to the CAM6 strain. Additionally, at pH 7, the CL4 strain showed a significant reduction (*p* < 0.05) in growth in comparison to the CAM6 strain, suggesting that the isolates have different pH sensitivities (Table 2). The inclusion of bile salts in MRS broth up to 0.15% stimulated the growth of the three stains. Nevertheless, for the highest concentration of bile salts (0.30%), CAM6 showed better growth than CAM7 and CL4.

The strains isolated from Zungo Pelado pigs were tolerant to high salt concentrations. All strains grew in NaCl concentrations up to 10%. While the CAM7 strain showed the highest growth at concentrations of up to 7% NaCl (*p* < 0.05), the CAM6 strain reached the highest level of growth at 10% bile salts. The incubation temperatures tested in our experiment did not modify the growth of *Lactobacillus plantarum* between groups (*p* > 0.05).

### 3.3. Assessment of Antimicrobial Activity of the Lactic Acid Bacteria Isolates

It was observed that all three selected isolates showed antagonistic activity against the different pathogens tested, except CAM7 for *Escherichia coli* and CL4 for *Klebsiella pneumoniae*. Specifically, *L. plantarum* CAM6 displayed the highest antagonistic activity against *E. coli* (19.7 mm) and *S. enterica* serovar Typhimurium (7.09 mm). Inhibition was also found for bacteria considered opportunistic, namely *Klebsiella pneumoniae* (8.20 mm) and *Pseudomonas aeruginosa* (9.90 mm) (Table 3).

### 3.4. Evaluation of the Lactic Acid Bacteria Isolates on Antibiotic Sensitivity Test

Selected strains were analyzed for their susceptibility to five antibiotics commonly used in the swine industry (Table 4). The three bacterial strains isolated from the Colombian native pig showed resistance to the antibiotics such as ciprofloxacin, tetracycline, doxycycline, and trimethoprim/sulfamethoxazole, with the singular exception of the CAM6 strain showing intermediate resistance to trimethoprim/sulfamethoxazole. However, all LAB strains were susceptible to amoxicillin.

## 4. Discussion

The objective of this study was to demonstrate whether LAB isolated from Colombian Creole pigs could be suitable probiotic candidates according to their in vitro characteristics. From a total of nine initial strains (before laboratory tests), only three viable strains were available at the beginning of the physiological characterization. They were Gram-positive/rod-shaped coccobacilli that were catalase- and oxidase-negative, confirming that they belonged to the LAB group.

The most common techniques we use to identify many facultative anaerobes are biochemical analyses, but these methods can yield varying results. In this study, we noted that biochemical characterization was not a more reliable identification approach despite being used as a primary method. For this reason, a molecular identification was necessary; in our case, all the strains were identified as *Lactobacillus plantarum*. Hence, genotypic systems are becoming valuable tools for use in studying a wide variety of microorganisms because they rely on comparisons of 16S rRNA sequences from unknown bacteria, are not sensitive to variable culture conditions, and provide more consistent results than the current standard microbial techniques [34].

However, issues with and limitations of this method have been observed. Species identification is based on the comparison of specific sequence homology with a known catalogue generated from previously identified organisms through conventional methodologies [35]. The leading molecular technology currently available for microbial identification is the sequence analysis of 16S rRNA, despite the availability of many new experimental molecular techniques for identification and the known problem of database accuracy and consistency over time [36]. These bacterial strains have been studied for their ability to form biofilms, a physical barrier that protects them from adverse environmental conditions [37] and makes them viable probiotic candidates for encapsulation.

Acid and bile salt tolerance are among the most important criteria for probiotic strain selection. All three isolates tolerated both pH 3 and 7. Interestingly, even though 16S rRNA identification showed the same identification profile, the isolates showed different growth rates in response to pH. Lactic acid bacteria are generally acidophilic, which means they are tolerant to low pH. In a condition of high free acid concentration (H+), free acids may cause growth inhibition [38]. Probiotic bacteria need to survive the passage through the stomach, where a pH of between 1.5 and 2.0 can be found [6], and into the intestinal tract [39].

Probiotic strains need to be tolerant to bile salts [1], and all three candidates showed good resistance up to a 0.15% concentration. In general, tolerance to bile salts is considered a condition for colonization and metabolic activity of bacteria in the host’s intestine. Consequently, when evaluating the potential use of LAB as a probiotic, it is important to evaluate their ability to tolerate bile salts [40]. The average concentration of bile salts in the small intestine is around 0.2 to 0.3% and may go up to 2% (*w*/*v*), depending upon the individual host and the type and amount of food ingested [41].

All strains were tolerant to high salt concentrations (up to 2%). These results suggest that the evaluated strains can withstand higher concentrations of NaCl without losing their growth capacity, which suggests that they can be used in starter crops in the dairy industry, as preservatives of meat and vegetables, and as probiotics [42].

The growth of the strains at 40 °C demonstrates the ability of these microorganisms to grow at extreme temperatures. Bacteria that exhibit this characteristic show higher growth rates and can proliferate in the gastrointestinal tract, where the temperature is higher than 37 °C [43]. Moreover, a high fermentation temperature reduces contamination by other microorganisms [44]. In addition, the capability of bacteria to grow at high temperatures is a favorable feature, allowing their use in technological procedures where high temperatures are applied [45].

*E.coli* and *S. enterica* serovar Typhimurium are the most commonly encountered pathogens in piglets responsible for neonatal diarrhea [46]. Antagonistic activity of *L. plantarum* against *E. coli* and *Salmonella* spp. in swine isolate was shown by Vera et al. [47]. Our results showed that the evaluated strains exerted antimicrobial activity, inhibiting microorganisms that are potentially pathogenic for animals. This inhibitory activity may be from the increase of primary metabolites such as ethanol, lactic acid, and carbon dioxide and the production of other antimicrobial compounds such as bacteriocins [31,48].

All isolates were resistant to ciprofloxacin, trimethoprim/sulfamethoxazole, tetracycline, and doxycycline but susceptible to amoxicillin. These results are in agreement with those of Jurado et al. [49], who also found sensitivity to penicillin in *L. plantarum*. Similarly, according to Shazali et al. [50], LAB isolated from broiler feces were found to be sensitive to penicillin and amoxicillin and resistant to ciprofloxacin and tetracycline.

Susceptibility to antimicrobial substances is an extremely vital selection property for a probiotic because this determines whether it can be coadministered with food supplements that contain antibiotics [51]. The high intrinsic resistance and susceptibility of microorganisms with probiotic activity to a range of antibiotics are quite significant. Since antibiotic resistance genes are generally carried by conjugative plasmids, they can be transferred to other bacteria [52], possibly resulting in antibiotic-resistant enteropathogenic bacteria. Therefore, it is also crucial to determine whether antibiotic resistance genes are present in chromosomes or plasmids [53].

## 5. Conclusions

In this study, the LAB isolates from the native Zungo Pelado breed of pigs showed good growth in response to pH 3.0 and higher concentrations (0.30%) of bile salts. In addition, the isolates possessed other desirable probiotic characteristics like tolerance to high NaCl concentrations (2 to 10%) and different temperatures (30, 37, and 42 °C). The isolated strains inhibited the in vitro growth of most of the pathogenic Enterobacteriaceae tested and were resistant to the presence of the antibiotics tested (except for CAM6, which showed intermediate resistance to trimethoprim/sulfamethoxazole), and all strains were susceptible to amoxicillin. The production of antibacterial compounds by these strains is an important characteristic to be studied later. The isolate CAM6, identified as *Lactobacillus plantarum*, showed consistent superiority over other isolates. Further studies to evaluate the effects of these probiotic candidate strains in commercial pigs are currently underway.

## Figures and Tables

**Table 1:** Comparisons between biochemical (API 50CHL) and 16S rRNA sequencing of the lactic acid bacteria isolates from the native Zungo Pelado breed of pigs.

Bacterial Strains	API 50 CH	GenBank Accession and URL
CAM6	*Lactobacillus plantarum*	MK523644.1; URL: https://bit.ly/2t4cHC9
CAM7	*Lactobacillus brevis*	MK537353.1; URL: https://bit.ly/2MPnwzd
CL4	*Lactobacillus acidophilus*	MK523645.1; URL: https://bit.ly/2tXJSYC

**Table 2:** In vitro assessment of functional properties of *Lactobacillus* spp. isolates from the native Zungo Pelado breed of pigs under various pH, bile salt, sodium chloride (NaCl), and temperature conditions (*n* = 3).

Items	Growth (Optical Density) of *Lactobacillus* Species			SEM ±	*p* Value
	CAM6	CAM7	CL4		
pH					
3	3.86	3.61	3.46	0.15	0.256
4	3.71	3.45	3.64	0.13	0.403
5.6	3.42 ^a^	3.04 ^b^	3.29 ^ab^	0.09	0.006
7	2.71 ^a^	2.45 ^ab^	2.14 ^b^	0.09	0.014
Bile salts (%)					
0.05	3.61	3.37	3.44	0.12	0.422
0.10	2.75 ^b^	3.06 ^ab^	3.28 ^a^	0.09	0.019
0.15	3.24	3.45	3.21	0.08	0.177
0.30	2.81 ^a^	2.71 ^a^	2.44 ^b^	0.05	0.006
NaCl (%)					
2	3.21	3.44	3.33	0.17	0.670
4	2.90 ^b^	3.35 ^a^	3.31 ^a^	0.08	0.015
7	2.54 ^b^	3.23 ^a^	2.12 ^c^	0.10	0.001
10	1.72 ^a^	1.12 ^b^	0.85 ^b^	0.12	0.006
Temperature (°C)					
30	3.70	3.38	3.52	0.18	0.506
37	3.57	3.48	3.51	0.17	0.935
42	2.47	2.89	2.86	0.14	0.136

^a,b,c^ Means within rows with different superscript letters are significantly different at *p* < 0.05; *n* = 3. SEM: Standard error of the mean.

**Table 3:** In vitro screening of antibacterial activity of *Lactobacillus* spp. isolates from the native Zungo Pelado breed of pigs against enteropathogenic bacteria (*n* = 3).

Pathogenic Bacteria	*Inhibition Halo* (mm)		
	CAM6	CAM7	CL4
*E. coli* strain NBRC 102203	19.7	0	6.9
*S. enterica* serovar Typhimurium 4.5.12	7.1	6.2	7.9
*Klebsiella pneumoniae* ATCC BAA-1705D-5	8.2	6.3	0
*Pseudomonas aeruginosa* ATCC 15442	9.9	9.7	7.3

**Table 4:** Evaluation of the antibiotic sensitivity of the *Lactobacillus* spp. isolates from the native Zungo Pelado breed of pigs (*n* = 3).

Strains	Antibiotics				
	Ciprofloxacin	Trimethoprim/Sulfamethoxazole	Amoxicillin	Tetracycline	Doxycycline
CAM6	8.1 (R)	19.5 (I)	24.3 (S)	8.2 (R)	8.2 (R)
CAM7	8.4 (R)	12.9 (R)	25.6 (S)	9.3 (R)	9.0 (R)
CL4	8.0 (R)	9.8 (R)	26.7 (S)	6.8 (R)	8.1 (R)
*E. coli* strain NBRC 102203	26.1 (S)	18.2 (I)	7.0 (R)	12.6 (R)	7.3 (R)
*S. enterica* serovar Typhimurium *4.5.12: i:-*	28.7 (S)	22.7 (S)	8.5 (R)	8.0 (R)	0 (R)
*K. pneumonia ATCC BAA-170* *5D-5*	0 (R)	8.8 (R)	0 (R)	0 (R)	0 (R)

Inhibition halo (mm) for each antibiotic was measured: ≥21 mm, susceptible (S); 16–20 mm, intermediate (I); ≤15 mm, resistant (R).
